# Lipids as *Trans*-Acting Effectors for α-Synuclein in the Pathogenesis of Parkinson’s Disease

**DOI:** 10.3389/fnins.2019.00693

**Published:** 2019-07-03

**Authors:** Kensuke Ikenaka, Mari Suzuki, Hideki Mochizuki, Yoshitaka Nagai

**Affiliations:** ^1^Department of Neurology, Graduate School of Medicine, Osaka University, Osaka, Japan; ^2^Department of Neurotherapeutics, Graduate School of Medicine, Osaka University, Osaka, Japan; ^3^Diabetic Neuropathy Project, Department of Sensory and Motor Systems, Tokyo Metropolitan Institute of Medical Science, Tokyo, Japan

**Keywords:** Parkinson’s disease, α-synuclein, lipid, fibril, synucleinopathy

## Abstract

Aggregation of α-synuclein (αSyn) plays a central role in the pathogenesis of Parkinson’s disease (PD) and dementia with Lewy bodies (DLB). Lewy bodies (LBs) and Lewy neurites, which consist mainly of aggregated αSyn, are widely observed in the affected regions of patient brains. Except for some familial forms of PD/DLB, most sporadic PD/DLB patients express the wild-type (WT) αSyn protein without any mutations, and the mechanisms as to how WT αSyn gains the propensity to pathologically aggregate still remains unclear. Furthermore, the mechanisms by which the same αSyn protein can cause different synucleinopathies with distinct phenotypes and pathologies, such as PD, DLB, and multiple system atrophy (MSA), still remain largely unknown. Recently, mutations in the *GBA1* gene (encoding glucocerebrosidase), which are responsible for the lysosomal storage disorder Gaucher disease (GD), have been reported to be the strongest risk factor for developing sporadic PD/DLB. We previously demonstrated that glucosylceramide accumulated by *GBA1* deficiency promotes the conversion of αSyn into a proteinase K-resistant conformation. Furthermore, decreased glucocerebrosidase activity has also been reported in the brains of patients with sporadic PD/DLB. Moreover, αSyn pathology has also been shown in the brains of lysosomal storage disorder patients, which show glycosphingolipid accumulation. These observations suggest the possibility that altered lipid metabolism and lipid accumulation play roles in αSyn aggregation and PD/DLB pathogenesis. Indeed, several previous studies have demonstrated that lipid interactions affect the conformation of αSyn and induces its oligomerization and aggregation. In this review, we will give an overview of the association between αSyn aggregation and lipid interactions from the viewpoints of the etiology, pathology, and genetics of PD/DLB. We also discuss the distinct species of αSyn aggregates and their association with specific types of synucleinopathies, and introduce our hypothesis that lipid interactions play a role as *trans*-acting effectors in producing distinct strains of αSyn fibrils.

## Introduction

Parkinson’s disease (PD) is a progressive neurodegenerative disorder characterized by preferential loss of dopaminergic neurons in the pars compacta of the substantia nigra (Scott and Netsky, 1961). PD is the second most common neurodegenerative disease after Alzheimer disease, and its prevalence is growing steadily. Clinical features of PD include motor symptoms, such as resting tremor, bradykinesia, and rigidity, as well as non-motor symptoms, such as dementia, depression, autonomic failure, and hallucinations (Marinus et al., 2018). Dopamine replacement therapy is widely used to improve the motor symptoms of PD patients, but does not attenuate disease progression. The exact mechanisms causing PD are still unknown, but the deposition of Lewy bodies (LBs) in the substantia nigra, which are composed mainly of fibrillar alpha-synuclein (αSyn), is a pathological hallmark of PD (Spillantini et al., 1997, 1998b). LBs are also widely observed in the cortex and other brain regions of patients with dementia with Lewy bodies (DLB) (Spillantini et al., 1998a), which predominantly exhibits dementia accompanied with frequent visual hallucinations and dopa-responsive Parkinsonism (Burkhardt et al., 1988; Byrne et al., 1989; Klatka et al., 1996). Since these diseases share pathological and clinical features, PD and DLB are considered to belong to the spectrum of disorders called Lewy body disease.

Genetic studies have demonstrated that missense or multiplication mutations in the αSyn gene cause familial forms of PD/DLB (Polymeropoulos et al., 1997), and that single nucleotide polymorphisms (SNPs) in the αSyn gene are major risk factors for sporadic PD/DLB (Satake et al., 2009; Simon-Sanchez et al., 2009). Considering the above pathological and genetic evidence, αSyn aggregation is a key target for developing disease-modifying therapies for PD/DLB and identifying the triggers of αSyn aggregation is expected to lead to new therapeutic strategies that attenuate or prevent PD/DLB.

Recently, there has been a large amount of discussion about the interaction of lipids with αSyn, which affects the structure of αSyn and accelerates its aggregation. In this review, we give an overview of the association between αSyn aggregation and lipid interactions from the viewpoints of the etiology, pathology, and genetics of PD/DLB patients and experimental models. We also propose our hypothesis that lipids interacting with αSyn may work as *trans*-acting effectors that induce variable conformational changes in the αSyn monomer, into structurally distinct αSyn fibrils, which may lead to clinically distinct synucleinopathies.

## α-Synuclein

### Physiological Characteristics of αSyn

**FIGURE 1:**
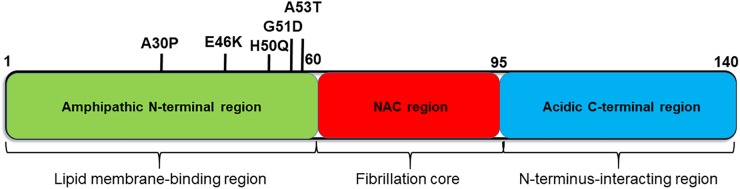
Diagram of the αSyn protein showing the characteristics of each region. αSyn can be divided into three regions; the N-terminal region (green), non-amyloid β-component (NAC) region (red), and C-terminal region (blue). The sites of causative mutations for familial PD are shown below, and functions and structural features are listed above each region.

αSyn is a small (14 kD) cytosolic protein that is enriched in presynaptic terminals. It consists of three major regions; the N-terminus (amino acid (aa) 1–60), the non-amyloid beta component (NAC, aa 61–95), and the C-terminus (aa 96–140) (Figure 1). In solution, αSyn is considered to be an intrinsically disordered protein, which lacks a single stable structure (Weinreb et al., 1996). Although the exact molecular function of αSyn has not yet been fully elucidated, it has been proposed to interact with biological membranes and membrane proteins, and to play roles in synaptic plasticity and neurotransmitter release (Chandra et al., 2004; Fortin et al., 2005; Nakata et al., 2012; Burre, 2015). In particular, αSyn was found to promote the assembly of SNARE complexes both *in vivo* and *in vitro* via the formation of multimers at the surface of synaptic vesicles (Burre et al., 2010).

### Role of Lewy Bodies in the Pathogenesis of PD/DLB

Classical LBs are round and eosinophilic cytoplasmic inclusions that displace other cytoplasmic components. They consist of a dense core surrounded by a halo of radiating fibrils with a width of 10 nm (Roy and Wolman, 1969; Spillantini et al., 1998b). Cortical LB is mainly found in the cortex of DLB patients and advanced PD patients and their morphologies are slightly different from classical LB, which are less defined and lack halos (Kosaka et al., 1976; Spillantini et al., 1998b). The components of LB are mainly αSyn, together with many other molecules, including proteins, such as neurofilament (Trojanowski and Lee, 1998), microtubule-associated protein 1B (Jensen et al., 2000), and galectin-3 (Flavin et al., 2017), as well as various lipids (Araki et al., 2015). The mechanism as to how LB is formed still remains unclear.

### Genetics of PD/DLB

Several point mutations in the αSyn gene are linked to autosomal-dominant PD/DLB (Polymeropoulos et al., 1997). Both pathogenic missense mutations (A53T, A30P, E46K, G51D, and H50Q) (PARK1) and multiplication of the entire gene (duplications and triplications) (PARK4) cause familial types of PD/DLB (Polymeropoulos et al., 1997; Kruger et al., 1998; Chartier-Harlin et al., 2004; Zarranz et al., 2004; Appel-Cresswell et al., 2013; Kiely et al., 2013). Moreover, in 2009, a European and a Japanese group both independently performed a genome-wide association study on sporadic PD and demonstrated strong associations of SNPs in the αSyn gene with PD (Satake et al., 2009; Simon-Sanchez et al., 2009), which have recently been shown to be also associated with DLB (Guerreiro et al., 2018). Taken together, these lines of genetic evidence for a causative role of αSyn as well as pathological evidence for the accumulation of αSyn in LBs strongly indicate the central role of αSyn in the pathogenesis of sporadic PD/DLB.

### αSyn Aggregation

As fibrillar αSyn is a major component of LB, the mechanism of fibril formation of αSyn has been studied extensively. Although αSyn is an intrinsically disordered protein, it forms a β-sheet-rich structure when aggregated (Maiti et al., 2004). Amyloid-like fibril formation of αSyn has been experimentally reproduced *in vitro*, and the resulting fibrils are morphologically similar to those found in LBs of patient brains (Rochet et al., 2000; Serpell et al., 2000).

Previous studies demonstrated that three major regions of αSyn, namely, the C-terminus, NAC, and N-terminus, play different roles in the process of fibril formation (Figure 1). First, the NAC region plays a central role in fibril formation and aggregation, via its formation of cross β-sheet structures. Indeed, several studies have shown that the NAC is necessary and sufficient for the aggregation and toxicity of αSyn (Giasson et al., 2001; Periquet et al., 2007; Rodriguez et al., 2015).

The negative charges at the C-terminal region of αSyn play an important role in inhibiting fibril formation, by interacting with the NAC region and masking it (Hong et al., 2011). The C-terminal region is also known as a metal-binding site. Upon binding to metals, such as Al^3+^, the C-terminal domain loses its inhibitory effect on NAC, leading to αSyn aggregation (Uversky et al., 2001; Dedmon et al., 2005; Ly and Julian, 2008). The C-terminus was also shown to form transient, long-range interactions with the N-terminus resulting in the formation of multiple compact monomeric structures (Bertoncini et al., 2005). These compact structures of αSyn are resistant to aggregation. Moreover, C-terminally truncated forms of αSyn were found to aggregate faster than the full-length protein (Hoyer et al., 2004; Li et al., 2005; Anderson et al., 2006). Interestingly, truncation of the C-terminal domain of αSyn has been detected in the brains of PD/DLB patients and a specific antibody against truncated αSyn stained the core of LBs (Zhang et al., 2017).

The N-terminal region of αSyn (aa 1–60) is the amphipathic region, and is involved in its interaction with lipid membranes and the structural formation of natively unfolded αSyn (George et al., 1995; Uversky, 2007). Seven imperfect repeats containing a KTKEGV consensus motif are found in the N-terminal region and in the NAC region close to the N-terminal region (aa 61–85), which play an important role in membrane binding (Jakes et al., 1994; Zarbiv et al., 2014). Similar motifs are also found in apolipoprotein, which is known to cause systemic amyloidosis (George et al., 1995). Very interestingly, all the missense mutations of aSyn are located in the N-terminal region, suggesting the importance of membrane binding for its inherent toxicity. In the next section, we will introduce and discuss the interaction of lipids with αSyn and their roles in αSyn aggregation.

## αSyn and Lipid Metabolism

### Lipid Membrane Binding and Structural Changes of αSyn

Although αSyn aggregation is a central player in PD/DLB pathogenesis, the mechanisms as to how wild-type (WT) αSyn without any mutations gains pathological aggregation properties in sporadic PD patients remain unknown. To solve this important question, many recent studies have examined the association between the nature of the interaction of αSyn with lipids and the propensity of αSyn to aggregate.

There are two possible mechanisms by which lipid membranes promote the aggregation of αSyn. One possibility is that the membrane surface facilitates the local concentration of αSyn. Consistently, the magnitude by which lipids affect the aggregation of αSyn was found to correlate with the strength of binding of αSyn to lipids by the following two main parameters: the lipid-to-protein-ratio and the chemical properties of the lipids (Zhu et al., 2003; De Franceschi et al., 2011; Galvagnion et al., 2015, 2016). The other mechanism is that membrane binding induces the formation of an “αSyn seed,” by changing the protein conformation of αSyn (Lee et al., 2002). αSyn is a intrinsically disordered protein that does not adopt a stable structure; however, it has been shown that upon binding to small unilamellar lipid vesicles or membranes, a fraction of the first 95 residues of αSyn undergoes a structural change from random coil to a horseshoe-like two-helix antiparallel α-helix (Chandra et al., 2003; Drescher et al., 2008; Bodner et al., 2009).

The precise mechanisms as to how membrane-bound α-helical αSyn changes its structure into a β-sheet and starts to aggregate is still unknown. However, it is well known that membrane lipids play an important role in this process, and therefore, alterations in the lipid composition of neuronal membranes may be a key step in the pathogenesis of PD.

### Glucocerebrosidase 1 (*GBA1*) Mutations and PD/DLB

One proof of concept that explains the involvement of lipid dysregulation in the pathogenesis of PD/DLB is that *GBA1* mutations increase the risk of PD/DLB (Tayebi et al., 2003; Goker-Alpan et al., 2004; Sidransky and Lopez, 2012). The *GBA1* gene encodes the lysosomal enzyme glucocerebrosidase (GCase), an enzyme involved in sphingolipid metabolism, catalyzing its conversion to glucose and ceramides. Homozygous mutations in the *GBA1* gene cause Gaucher disease (GD), which is the most common lysosomal storage disorder. The accumulation of glucosylceramide (GlcCer) in macrophages is observed as “Gaucher cells,” which serve as the hallmark of GD.

Interestingly, a subset of type 1 GD patients was reported to demonstrate typical PD symptoms (Neudorfer et al., 1996). A multicenter genetic analysis confirmed that heterozygous mutations in the *GBA1* gene are significant risk factors for PD (Sidransky et al., 2009) and also for DLB (Nalls et al., 2013; Gamez-Valero et al., 2016). Clinical studies reported that GBA1-linked PD/DLB is virtually indistinguishable from idiopathic PD/DLB, with a slightly earlier age of onset (Nichols et al., 2009; Gamez-Valero et al., 2016) and higher prevalence of cognitive impairment (Sidransky et al., 2009). Regarding pathology, no differences in LB pathology have been reported between GBA1-linked PD and idiopathic PD (Neumann et al., 2009).

Importantly, not only a rare mutation in the *GBA1* gene increases the risk of developing PD/DLB, but also a decrease in GCase activity has been reported in the biofluid or brains of sporadic PD patients (Balducci et al., 2007; Gegg et al., 2012; Parnetti et al., 2014; Chiasserini et al., 2015; Rocha et al., 2015; Moors et al., 2019). Furthermore, a decrease in GCase activity in the brains of sporadic PD/DLB patients was reported to be associated with an increase in the amounts of insoluble αSyn (Murphy et al., 2014). These findings indicate the possibility that decreased GCase activity is a common pathway leading to αSyn aggregation in the brain (Gegg and Schapira, 2018).

### Glucosylceramide and αSyn Aggregation

How does decreased GCase activity lead to αSyn aggregation? Several *in vitro* studies have shown that both GlcCer and glucosylsphingosine can directly cause monomeric αSyn to aggregate (Mazzulli et al., 2011; Taguchi et al., 2017; Maor et al., 2019). We showed a significant increase in αSyn dimers upon incubation with GlcCer-containing liposomes, which is consistent with the finding that the amount of αSyn dimers was significantly increased in erythrocytes of GD patients (Argyriou et al., 2012; Suzuki et al., 2015; Moraitou et al., 2016).

From the view of a loss-of GCase function model, Mazzulli et al. (2011) showed that GCase deficiency leads to the accumulation of GlcCer in neurons, which in turn promotes the formation of toxic αSyn aggregates in cultured neuronal cells. Interestingly, not only GlcCer directly affects the amyloid formation of purified αSyn by stabilizing soluble oligomeric intermediates, but αSyn also inhibits the lysosomal activity of normal GCase, suggesting a bidirectional association between αSyn and GCase in the pathogenesis of the synucleinopathies (Mazzulli et al., 2011). We hence examined the effects of GCase deficiency on the neurotoxicity of αSyn in a *Drosophila* model (Suzuki et al., 2015). Behavioral and histological analyses showed that knockdown of the *Drosophila* homologue of *GBA1* exacerbates locomotor dysfunction and loss of dopaminergic neurons of αSyn-expressing flies. We showed that GlcCer accumulated owing to GCase deficiency interacts with αSyn and confers proteinase K (PK)-resistance to αSyn, suggesting that GlcCer promotes the toxic conformational conversion of αSyn.

### Lipid Dysregulation in Sporadic PD/DLB

What is the status of lipid metabolism in sporadic PD/DLB patients? As discussed above, decreased activity of the lysosomal enzyme GCase has been reported in the brains of sporadic PD/DLB patients, suggesting the accumulation of glycosphingolipids and αSyn (Gegg et al., 2012; Moors et al., 2018). Indeed, several studies reported altered lipid metabolism in PD/DLB patients. For example, plasma ceramides and monohexosylceramides were increased in DLB patients (Savica et al., 2016) and polyunsaturated fatty acids were increased in PD/DLB patient brains (Sharon et al., 2003).

Interestingly, the level of lysosomal enzyme activity was reported to be higher in the substantia nigra, and selectively decreased in older people as well as in PD/DLB patients, suggesting a possible explanation as to how PD/DLB pathology can selectively affect specific regions of the brain (Murphy et al., 2014; Chiasserini et al., 2015). Considering that lipid accumulation leads to αSyn aggregation, lipids might be accumulated in LBs in the brains of PD/DLB patients. Indeed, we examined LBs in the brains of PD patients by Fourier transform infrared microscopy, and found that lipids are accumulated in the core of LBs and are surrounded by a halo that is rich in fibril-like β-sheet structures (Araki et al., 2015). These results are consistent with a report that the core of LB was stained with the lipid-soluble fluorescent dye rhodamine B (Issidorides et al., 1991).

## Structural Variants of αSyn and Lipids

### Clinical and Pathological Diversity of Synucleinopathies

αSyn is aggregated and accumulated in a group of neurodegenerative diseases, including PD, DLB, and multiple system atrophy (MSA), which are collectively known as the synucleinopathies. Although they share the same disease-causing protein, each synucleinopathy demonstrates distinct clinical and pathological phenotypes, which may result from diverse pathological αSyn strains in patients (Guo et al., 2013; Prusiner et al., 2015). Recent studies have indicated that αSyn may act in a way similar to prions and that structural variants of αSyn aggregates may behave as strains with distinct biochemical and functional properties, inducing specific phenotypic traits, which might finally provide an explanation for the clinical heterogeneity observed among PD, DLB, and MSA patients (Peng et al., 2018b). In this section, we summarize the characteristics of different αSyn strains that are experimentally generated from recombinant αSyn monomers. We also discuss αSyn strains that may potentially exist in synucleinopathy patients. Finally, we describe the possible roles of lipids in determining the structural diversity of αSyn fibrils.

### Distinct Species of αSyn Fibrils

**FIGURE 2:**
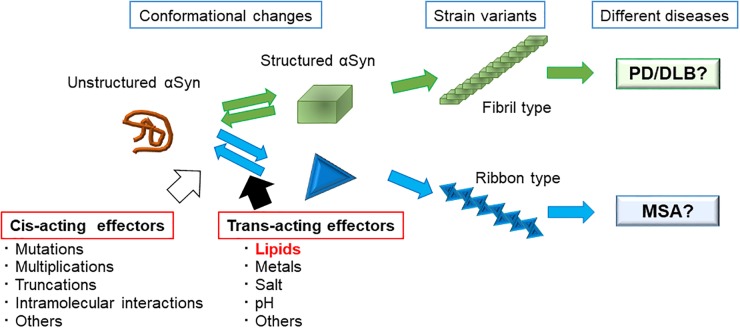
Strain variants of αSyn leading to different clinical phenotypes and possible factors that affect αSyn assembly. We propose the possible mechanisms as to how different αSyn species cause the different types of synucleinopathies. Unstructured αSyn can adopt several structural conformations. Once αSyn forms a structure that favors assembly with each other, it forms fibrils. The final morphology and characters of the fibrils are strongly associated with the initial conformational change. As changing the buffer conditions, such as adding salt or desalting leads to the formation of different types of fibrils, many environmental factors (*trans*-acting effectors) and some autologous factors (*cis*-acting effectors) are thought to affect the type of fibril that is formed.

The diversity of αSyn fibril structures have been well studied using αSyn mutants that cause familial forms of PD (Serpell et al., 2000; Choi et al., 2004; Sahay et al., 2015). The mutations A53T and E46K have been shown to significantly accelerate fibrillation *in vitro* when compared with WT αSyn, and the morphologies are also distinct from WT fibrils; A53T and E46K αSyn form twisted fibrils 5–14 nm in width, whereas WT αSyn forms non-twisted fibrils. The morphologies of A30P and G51D αSyn fibrils appears to be similar to those formed from WT fibrils, with a filament width of 6–9 nm (Serpell et al., 2000). In another study using single-molecule fluorescence, Sierecki et al. (2016) reported that pathogenic αSyn mutants surprisingly segregated into two groups: one group (E46K, H50Q, and A53T), formed large aggregates and fibrils whereas the other group (WT, G51D, and A30P) tended to form mostly oligomers. These findings suggest that *cis*-acting effects of mutations on αSyn itself lead to the distinct species of fibrils (Figure 2).

How about the *trans*-acting effectors that affect the fibrillation of WT αSyn? In 2013, Bousset et al. (2013) assessed the effects of different buffer conditions, i.e., with or without salt, on the formation of αSyn fibrils, and showed that different conditions created two strains with different structures, levels of toxicity, and *in vitro* and *in vivo* seeding and propagation properties. Later, in 2015, they showed that distinct αSyn strains demonstrated different seeding capacities, inducing strain-specific pathologies and neurotoxic phenotypes *in vivo* (Peelaerts et al., 2015). These discoveries led to the hypothesis that *trans*-acting effectors affect αSyn fibrillation processes and that distinct strains may account for different clinicopathological traits among the synucleinopathies (Figure 2).

### Cellular Environment and Synuclein Species

To date, there have been no direct lines of evidence clearly showing the detailed conformational differences among αSyn species in PD, DLB, and MSA. However, several reports strongly indicate the existence of different αSyn species in the different synucleinopathies. For example, the solubility of αSyn in the brains of MSA patients is very different from that in the brains of PD/DLB patients. αSyn extracted from MSA patient brains mainly appears in the sodium dodecyl sulfate (SDS)-soluble fraction, whereas αSyn extracted from PD/DLB patient brains appears in the insoluble fraction (Campbell et al., 2001). Moreover, Hirohata et al. (2011) reported that αSyn fibrils amplified from the cerebrospinal fluid of MSA patients by protein misfolding cyclic amplification using recombinant αSyn showed different widths compared with fibrils amplified from PD and DLB patients. In 2015, Prusiner et al. (2015) reported that brain lysates from MSA patients containing an abnormal form of αSyn could transmit this abnormal conformation to transgenic mice expressing αSyn 140^∗^A53T; however, neither αSyn from normal control brain lysates nor PD brain samples were able to transmit an abnormal conformation.

Then, what are the *trans*-acting effectors and how are different strains created in the brains of patients? Recently, Peng et al. proposed the hypothesis that different cellular environments of neurons and oligodendrocytes promote the formation of distinct αSyn strains (Peng et al., 2018a). They showed that oligodendrocytes but not neurons transform misfolded αSyn into a strain that has more resistance to PK and stronger transmissibility, which is similar to fibrils obtained from MSA patients, thus highlighting the fact that distinct αSyn strains are generated by different intracellular milieus.

### Possible Roles of Lipid Diversity in Synucleinopathies

Considering the types of cellular milieus that may differ between neurons and oligodendrocytes, we believe that lipid components are a possible candidate (Figure 2). The brain is primarily comprised of lipid, where lipids represent up to 50% of its dry weight. Some lipids are found abundantly in the brain, such as galactosylceramide (GalCer) and sulfatide in myelin, and ganglioside GM1 in neurons, and lipids are not homogeneously distributed in the brain. In fact, glial cells and neurons have specific glycosphingolipid compositions (van Echten-Deckert and Herget, 2006). Oligodendrocytes mainly express GalCer, sulfatide, and GM3, whereas astrocytes highly express ganglioside GM3. Neurons contain the widest variety of gangliosides, including GM1, GD1a, GD1b, and GT1b (van Echten-Deckert and Herget, 2006). Taking these observations into consideration, the high content of glycosphingolipids in brain membranes have a considerable effect on the aggregation process of the αSyn in each neuronal cell type. Indeed, our *in vitro* results suggest the possibility that distinct prion-like αSyn strains are generated by GlcCer and GM1 (Suzuki et al., 2015, 2018). The PK-digested band pattern of αSyn, which indicates structural differences of various amyloid fibrils, was different between αSyn incubated with GlcCer-containing liposomes and αSyn incubated with GM1-containing liposomes (Suzuki et al., 2015). We therefore propose that lipid components act as *trans*-acting effectors for αSyn in the pathogenesis of PD (Figure 2).

Few studies to date have investigated the changes in lipid metabolism of MSA patients. Don et al. (2014) analyzed the amount of galactosphingolipids in the white matter of the brains of MSA patients. They showed that total sphingomyelin, sulfatide, and GalCer levels were selectively decreased in disease-affected white matter (primary motor cortex), whereas no significant differences were detected in unaffected white matter (visual cortex). Interestingly, the physiological expression of these lipids are higher in the motor cortex than in the visual cortex, suggesting a possible explanation as to how αSyn selectively accumulates in specific regions of the brain in MSA patients.

## Conclusion

In this review, we gave an overview of αSyn aggregation in PD/DLB and the possible roles of lipid dysregulation in the PD/DLB pathogenesis. Many genetic and pathological studies have shown that lipid metabolism is affected in the brains of PD patients, and many *in vitro* and *in vivo* studies have suggested that this might initiate conformational changes in αSyn, leading to its aggregation. Some preliminary data suggest the possibility that the accumulated lipids not only accelerate αSyn aggregation but also determine the different species of αSyn fibrils. If our hypothesis can be proven, we will be able to extend our understanding of the distinct pathologies among the synucleinopathies, such as PD, DLB, and MSA, and why they show different clinicopathological features despite showing accumulation of the same WT αSyn. Further studies, such as a comparison of lipid metabolism in the brains or biosamples of PD/DLB and MSA patients and investigations on how such altered lipid metabolism affects αSyn aggregation, are required to show the association between altered lipid metabolism and different αSyn species in PD/DLB and MSA.

## Author Contributions

KI wrote the first draft of the manuscript. All authors contributed to manuscript revision, read it, and approved the submitted version.

## Conflict of Interest Statement

The authors declare that the research was conducted in the absence of any commercial or financial relationships that could be construed as a potential conflict of interest.
